# Supplementary material for: Single-cell and spatial transcriptome characterize coinhibitory cell-cell communications during histological progression of lung adenocarcinoma
Source: Front Immunol. 2024 Oct 15;15:1430163. doi: 10.3389/fimmu.2024.1430163 (PMC11518759; doi:10.3389/fimmu.2024.1430163)
Supplement: Supplementary file 1 [file DataSheet1.pdf]

Single-cell and spatial transcriptome characterize coinhibitory cell-cell communications during histological progression of lung adenocarcinoma

Judong Luo, Qianman Gao , Meihua Wang, Hong Zhu*, Hui Liu*

Supplementary Figures

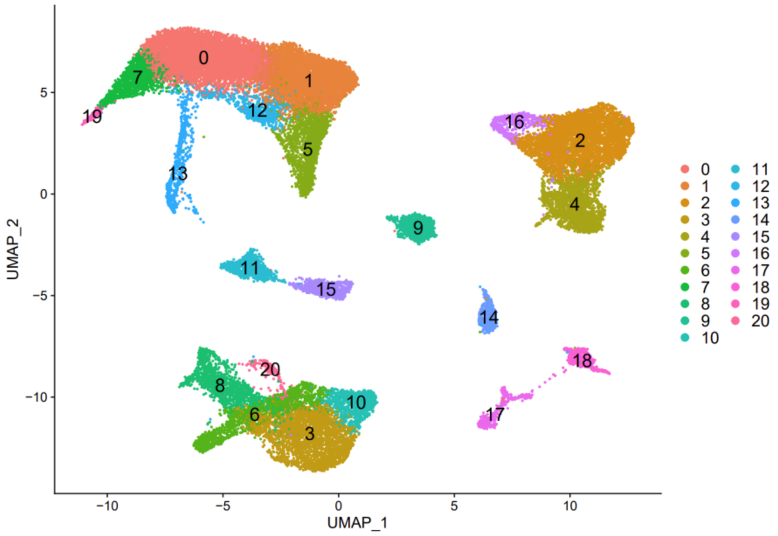

Figure S1. Single cells from six LUAD samples were stratified into 21 cell clusters using the graph-based Louvain clustering algorithm

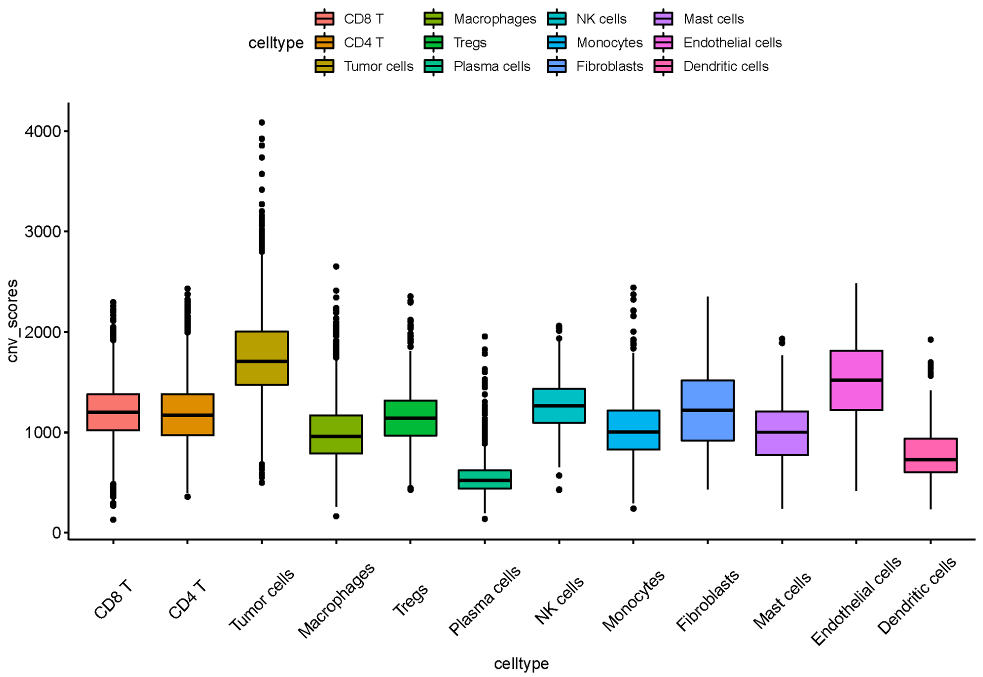

Figure S2. Box plots showed the CNV scores for each cell type calculated from inferCNV algorithm

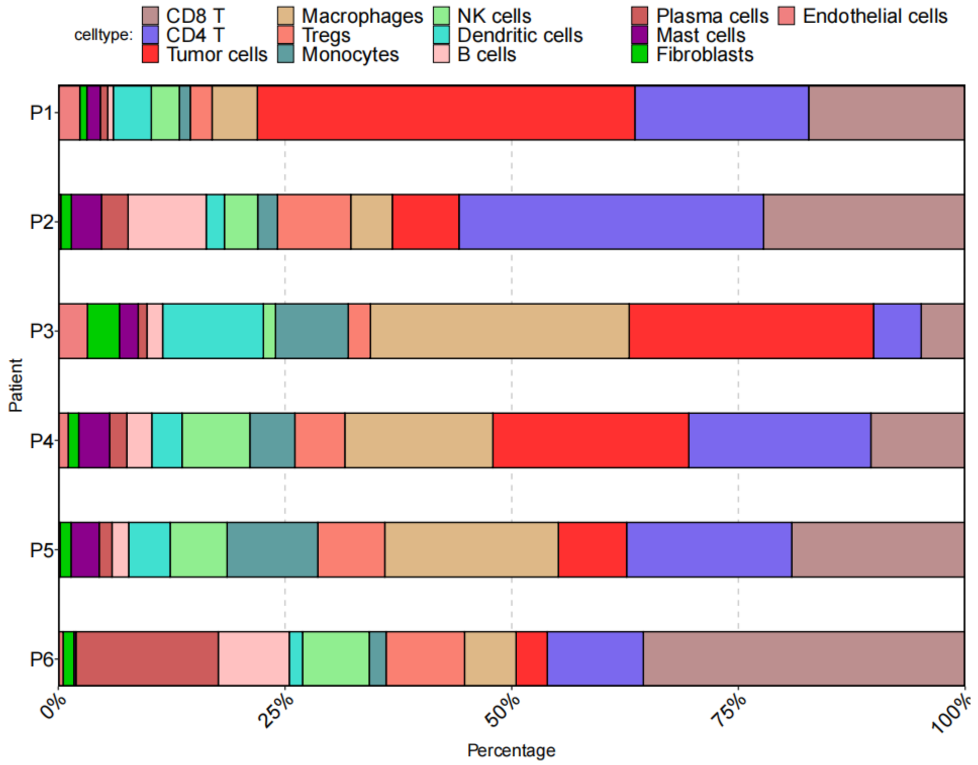

Figure S3. Proportions of cell compositions of six LUAD patients

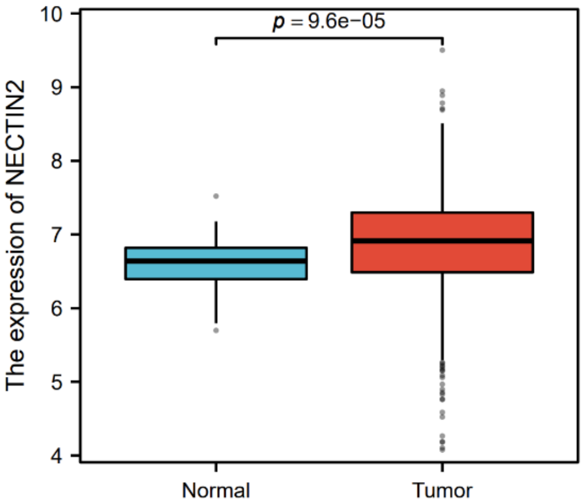

Figure S4. Expression of NECTIN2 gene in normal tissues and tumor tissues of TCGA-LUAD cohort

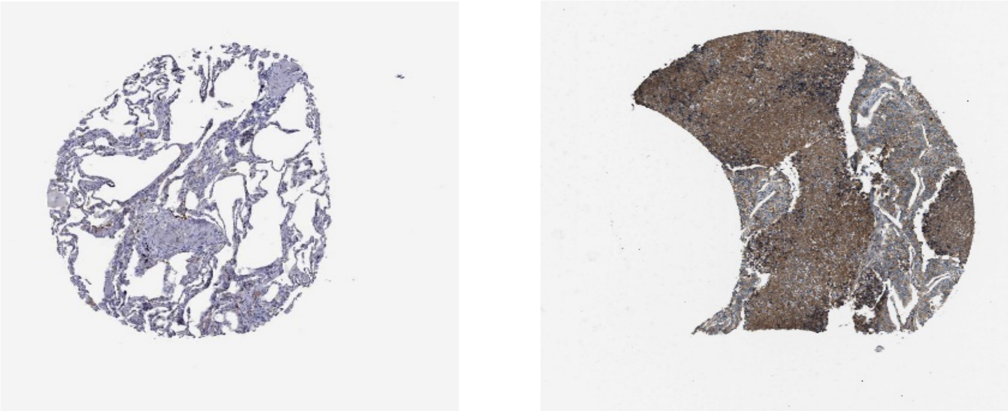

Figure S5. Immunohistochemistry of PVR gene in normal (left) and tumor (right) tissues of lung adenocarcinoma patients from HPA database

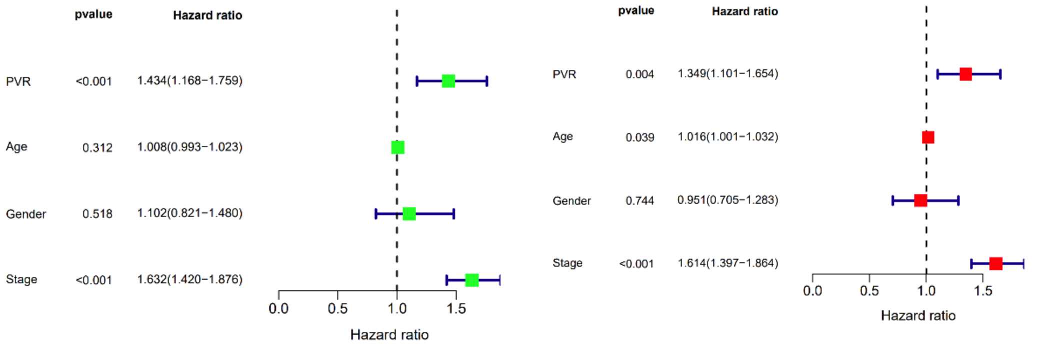

Figure S6. Univariate (left), multivariate (right) Cox regression analysis using PVR gene and other clinical variables

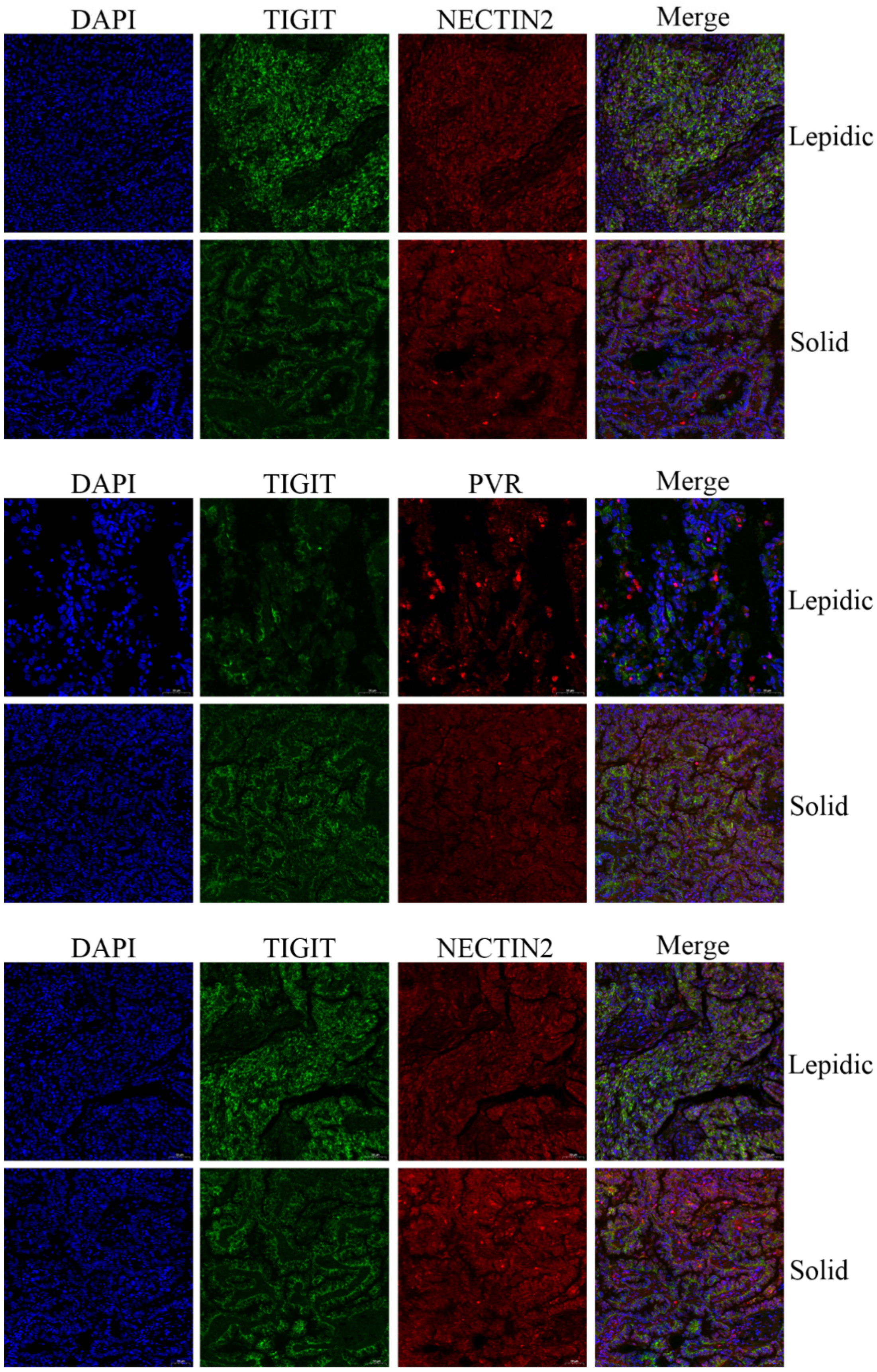
DAPI
TIGIT
NECTIN2
Merge
Lepidic
Solid
DAPI
TIGIT
PVR
Merge
Lepidic
Solid
DAPI
TIGIT
NECTIN2
Merge
Lepidic
Solid

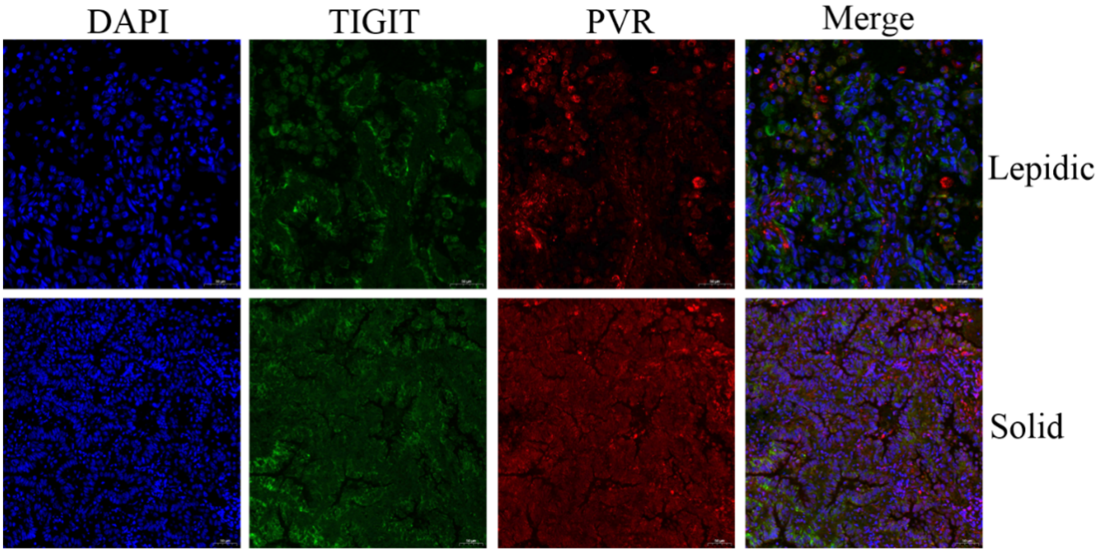

Figure S7. Immunofluorescence staining of TIGIT, NECTIN2 and PVR proteins in tumor tissues from four patients with two histologic patterns (Scale bar = 50 μm).

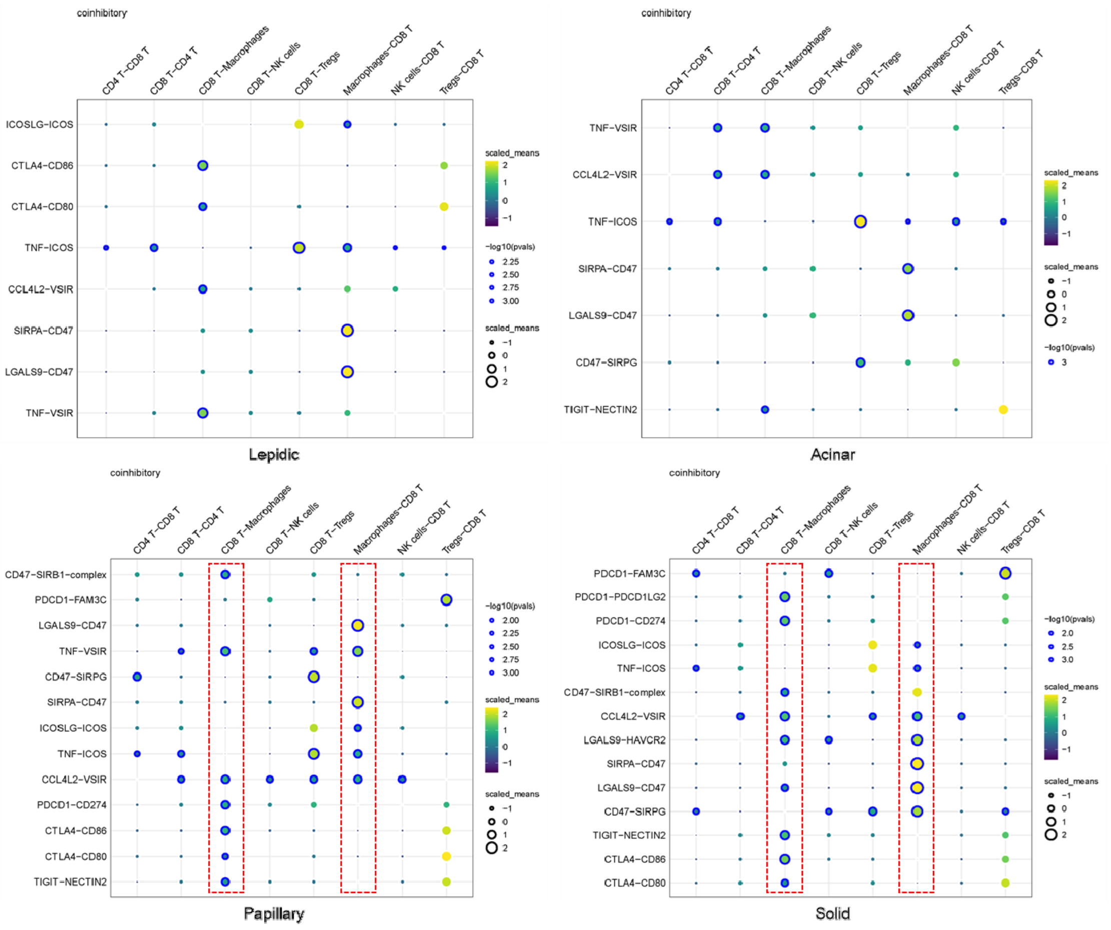

Figure S8. Co-inhibitory interactions of CD8+T cell and other types of immune cells in four histologic patterns, where each row represents receptor-ligand pairs, each column represents a pair of interacting cell types.

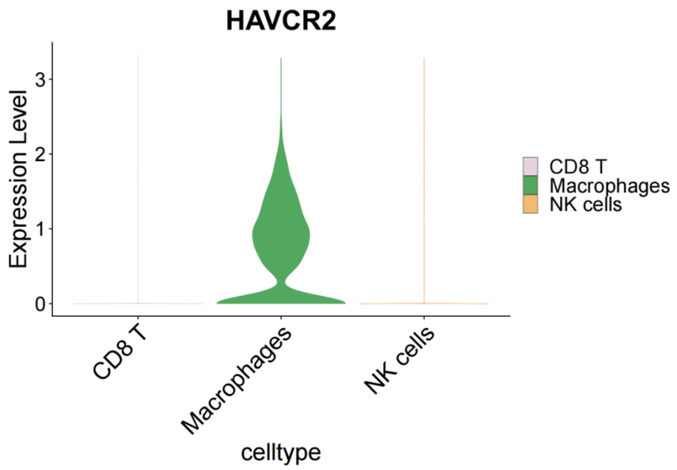

Fig.S9 HAVCR2 is highly expressed in macrophages but nearly absent in CD8+ T cells and NK cells

(a) Lepidic

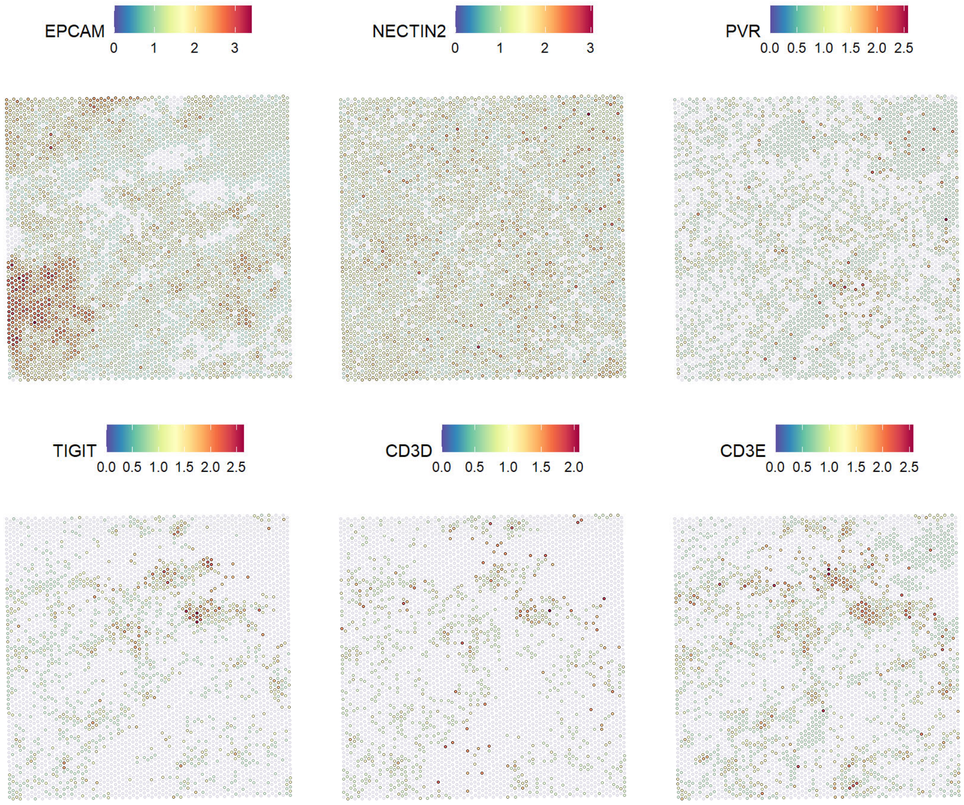

(b) Solid

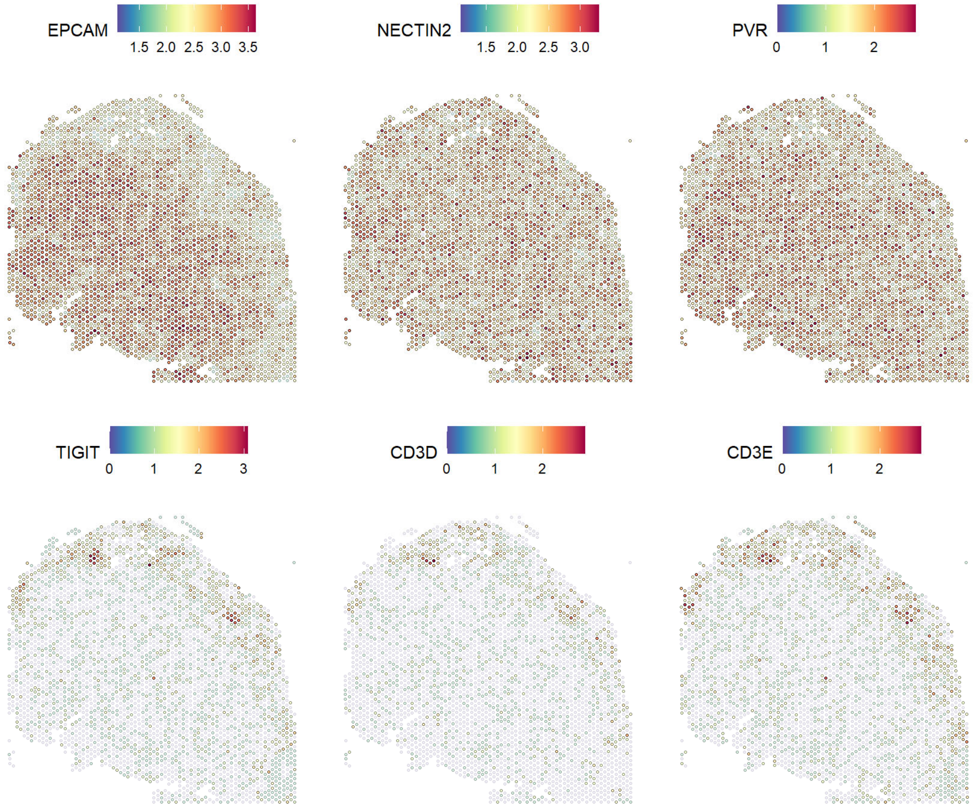

Figure S10 Spatial expression pattern of TIGIT correlated to T cell marker genes CD3D and CD3E in LUAD samples with lepidic (a) and solid (b) histologic patterns

Supplementary Tables

Table S1 Results of Univariate and multivariate regression for co-inhibitory genes

Gene	Univariate analysis		Multivariate analysis	
	Hazard ratio (95% CI)	P value	Hazard ratio (95% CI)	P value
**NECTIN2**	**1.380 (1.138 - 1.674)**	**0.001**	**1.360 (1.118 - 1.653)**	**0.002**
TIGIT	0.885 (0.760 - 1.030)	0.115	-	-
HAVCR2	0.932 (0.823 - 1.057)	0.272	-	-
LGALS9	1.002 (0.880 - 1.142)	0.972	-	-
PDCD1	0.999 (0.872 - 1.146)	0.994	-	-
CD274	1.021 (0.917 - 1.136)	0.710	-	-
**CD80**	**0.817 (0.675 - 0.988)**	**0.037**	0.840 (0.692 - 1.020)	0.079
FAM3C	1.032 (0.895 - 1.191)	0.661	-	-
LAG3	0.987 (0.874 - 1.115)	0.834	-	-
FGL1	0.993 (0.937 - 1.052)	0.801	-	-
CD47	0.859 (0.728 - 1.015)	0.073	-	-
SIRPG	0.893 (0.770 - 1.035)	0.132	-	-
